# Case Report: A novel heterozygous variant of the *TCOF1* gene in Treacher Collins syndrome

**DOI:** 10.3389/fped.2025.1615309

**Published:** 2025-09-16

**Authors:** Lijuan Zhang, Fei Wang, Yanfang Zhu, Hongxiao Zhang, Yahong Liu

**Affiliations:** Department of Pediatrics, The Second Hospital of Lanzhou University, Lanzhou, China

**Keywords:** Treacher Collins syndrome, *TCOF1* gene, variant, treacle protein, case report

## Abstract

Treacher Collins syndrome (TCS) is a craniofacial malformation caused by the abnormal development of the first and second pharyngeal arches during embryogenesis. While pathogenic variants in *POLR1B*, *POLR1C*, and *POLR1D* are implicated, the *TCOF1* gene represents the primary causative locus. This case report describes a novel heterozygous frameshift variant, *TCOF1*:NM_001135243.2:c.3559delG (p.Ala1187GlufsTer21), identified in a child with TCS. This truncation disrupts the C-terminal nuclear localization signal (NLS), specifically ablating a phosphorylation site at residues 1,199–1,200. Consequently, ribosome biosynthesis and craniofacial neural crest cell development are impaired, culminating in characteristic clinical manifestations, including downslanting palpebral fissures, depressed nasal bridge, marked malar hypoplasia, mandibular hypoplasia, and microtia. Although the mother carried the same variant, she exhibited no clinical symptoms, suggesting incomplete penetrance. This variant is the first internationally reported instance. Its identification reinforces the central pathogenic role of *TCOF1* in TCS, underscores the functional significance of the treacle protein's NLS, and expands the variant database. Penetrance variability complicates genetic counseling, necessitating future research into its genetic characteristics to enhance prenatal diagnostic accuracy.

## 1 Introduction

Treacher Collins syndrome (TCS, OMIM 154500) is a rare developmental disorder with an incidence of 1:50,000 (1). It arises from the aberrant development of the first and second pharyngeal arches during the 5th to 8th gestational weeks. The major clinical characteristics encompass micrognathia, retrognathia, lower eyelid coloboma (with loss of medial eyelashes), microtia or anotia, external auditory canal atresia or stenosis, downslanting palpebral fissure secondary to lateral orbital hypoplasia, a large or protruding nose, and zygomatic bone hypoplasia. Minor features include a cleft lip with or without a concomitant cleft palate, preauricular hair displacement, airway obstruction, and conductive hearing loss due to middle and external ear malformations (2). TCS is typically diagnosed clinically at birth, supported by radiographic imaging and confirmed by molecular genetic testing. Management necessitates a staged, multidisciplinary approach across the patient’s lifespan.

TCS syndrome is genetically heterogeneous. The predominant gene involved is *TCOF1*, with additional contributions from *POLR1B*, *POLR1C*, and *POLR1D*. These genes exhibit distinct inheritance patterns: *TCOF1* and *POLR1B* variants are autosomal dominant, *POLR1C* variants are autosomal recessive, and *POLR1D* variants can be either autosomal dominant (AD) or recessive (3).

As the primary pathogenic locus for TCS, *TCOF1* is located at chromosome 5q32-q33.1. This gene comprises 28 exons and can encode the nucleolar phosphorylated Treacle protein (1,488 amino acids, 152 kDa) (4). Truncating variants in this gene lead to Treacle haploinsufficiency through three primary mechanisms: premature translational termination, impaired nucleolar localization, and compromised ribosomal RNA biogenesis. This functional impairment of Treacle disrupts neural crest cell (NCC) development, leading to craniofacial hypoplasia. Notably, genotype-phenotype associations remain contentious, and incomplete penetrance is mechanistically unexplained, implicating genetic modifiers or epigenetic regulators. Herein, we report a novel truncating *TCOF1* variant, *TCOF1*:NM_001135243.2:c.3559delG (p.Ala1187GlufsTer21), in a classical TCS proband. The variant exhibits incomplete penetrance, as it was inherited from an unaffected mother. This case adheres to the CARE reporting checklist.

## 2 Case description

### 2.1 Clinical characteristics

The proband was vaginally delivered at term (40 weeks) to non-dysmorphic consanguineous Chinese parents (21 March 2023), with no history of perinatal asphyxia. He was their second child. The first pregnancy was complicated by sudden intrauterine fetal demise at 32 weeks of gestation. The gross morphological assessment identified ear malformations and mandibular hypoplasia (no ancillary testing or autopsy was conducted). The proband's birth weight was 3.5 kg (within ±1 SD) and length was 50 cm (within ±1 SD). He was admitted to the neonatology unit due to severe choking episodes during oral feeding.

A physical examination revealed normal skin and hair pigmentation, alongside the following characteristic craniofacial dysmorphisms: downslanting palpebral fissures, depressed nasal bridge, marked malar hypoplasia, mandibular hypoplasia, right microtia, and left external auditory canal atresia with preserved pinna architecture. Additional findings included a grade I cleft palate and a continuous murmur at the left second intercostal space. Limb and abdominal examinations were unremarkable. Laboratory tests (complete blood count, biochemistry) were normal. Echocardiography confirmed patent ductus arteriosus (PDA). The infant received symptomatic and supportive care and the parents received feeding guidance before discharge at parental request.

At the 1-month visit (28 April 2023), his weight was 4.2 kg (within ±1 SD) and length 54.5 cm (within ±1 SD). At the 6-month visit (7 October 2023), the patient’s Gesell developmental quotient (DQ) scores indicated developmental delay (defined as DQ < 75): gross motor was 41.5 (moderate deficiency), fine motor was 37.7 (severe deficiency), adaptive behavior was 56.6 (mild deficiency), language was 50.9 (moderate deficiency), and personal-social was 50.9 (moderate deficiency). Auditory brainstem response (ABR) thresholds [right ear: 65 dB normal hearing level (nHL); left ear: 55 dB nHL] suggested asymmetric hearing loss.

**Figure 1:**
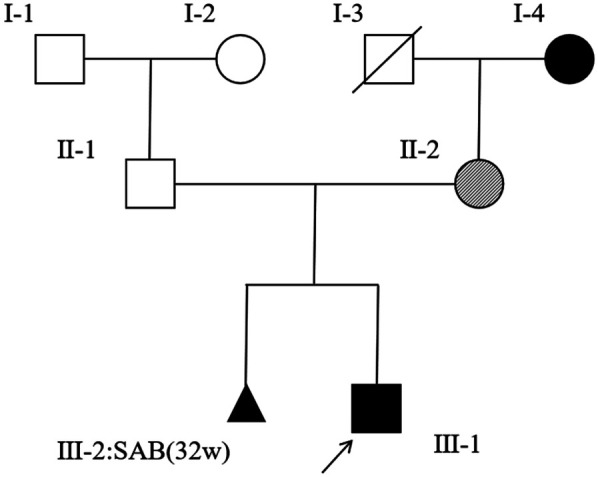
A pedigree of the family of the patient (Ⅲ-1). Squares indicate males, and circles indicate females. A black arrow represents affected subjects, clear symbols represent unaffected subjects, and crossed symbols represent deceased family members. Dashed circles indicate asymptomatic females carrying the causative variant. The aborted fetus is represented by a triangle.

Given the proband's clinical features (craniofacial dysmorphism, palatal cleft, developmental delay, congenital heart malformations, and concomitant hearing abnormalities) and a family history (Figure 1) of auricular deformities and hearing impairment in the maternal grandmother, TCS was strongly suggested. Hearing aids were fitted after an otolaryngology consultation. Rehabilitation therapy was initiated. Genetic testing was recommended, but was deferred by the family due to the infant's age.

**Figure 2:**
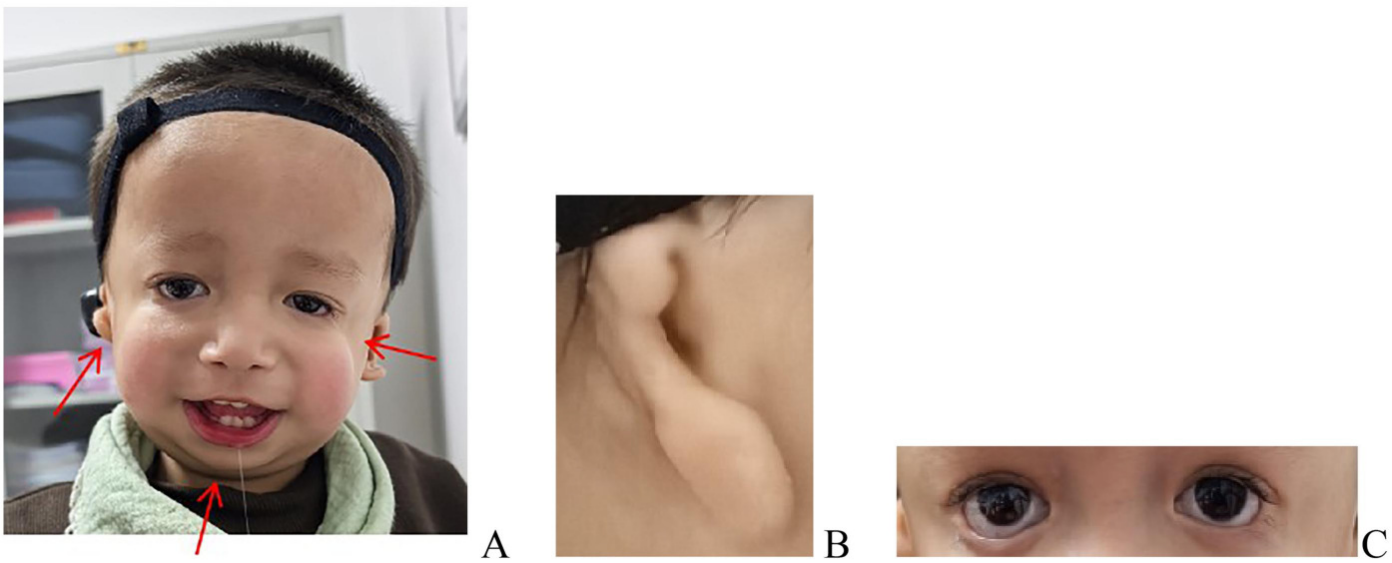
Dysmorphic features, including downslanting palpebral fissures, depressed nasal bridge, marked malar hypoplasia, mandibular hypoplasia, right microtia, and left external auditory canal atresia with preserved pinna architecture. **(A)** Overall appearance presentation. **(B)** Microtia. **(C)** Downslanting palpebral fissures and depressed nasal bridge.

**Figure 3:**
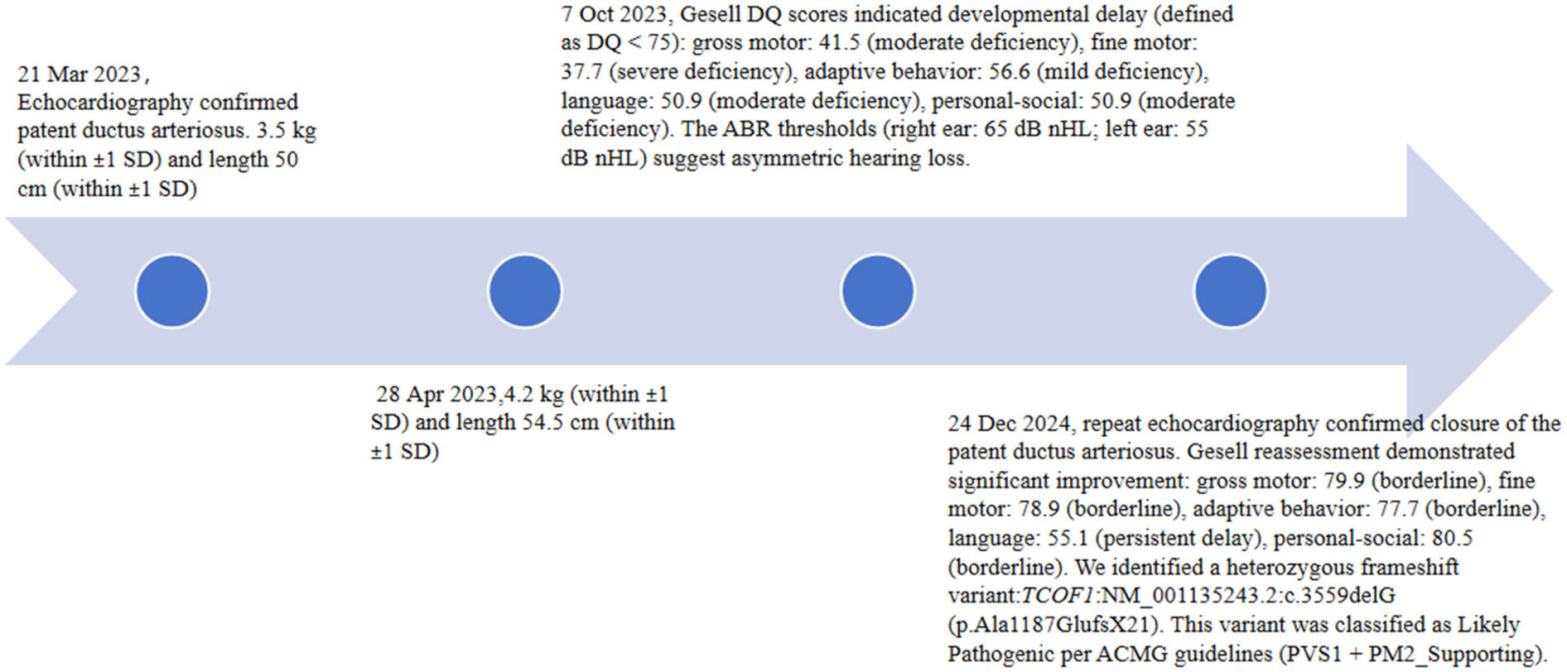
The timeline of this case.

At 1 year 8 months of age (24 December 2024), repeat echocardiography confirmed closure of the PDA (Figure 2). The patient’s Gesell DQ scores showed significant improvement with a gross motor score of 79.9 (borderline), fine motor score of 78.9 (borderline), adaptive behavior score of 77.7 (borderline), language score of 55.1 (persistent delay), and personal-social score of 80.5 (borderline). Following counseling regarding the infant's condition, the parents consented to genetic testing. The proband continues to receive rehabilitative therapy with regular auditory-verbal assessments. A staged surgical plan is proposed: palatoplasty beyond age 2, autologous microtia reconstruction in childhood, and orthognathic surgery in adolescence, supplemented by psychosocial support. The timeline is as shown in Figure 3.

### 2.2 Variant sequencing

Genomic DNA was extracted from the proband's blood using the QIAamp DNA Blood Mini Kit (Qiagen) per the manufacturer's instructions. DNA quality was assessed using a Nanodrop 2000. After repairing the fragment ends and adding A-tails, DNA libraries were prepared by ligating adapters to the fragments. Whole-exome sequencing (WES) was performed on the proband's DNA, with exome enrichment conducted using the GenCap-WES Capture Kit. Labeled probes were hybridized in the liquid phase, followed by linear PCR amplification of the libraries. Library quality was assessed, and after passing QC, sequencing was carried out on the Illumina NextSeq500 platform (5, 6).

The WES reads were aligned to GRCh37.p10 using the Burrows–Wheeler Aligner (BWA). The BAM files underwent duplicate marking using GATK’s MarkDuplicates tool and base quality score recalibration using GATK’s Base Recalibrator tool. Finally, GATK’s HaplotypeCaller tool processed the Recal BAM to detect the single-nucleotide polymorphisms (SNPs) and deletions (indels).

**Figure 4:**
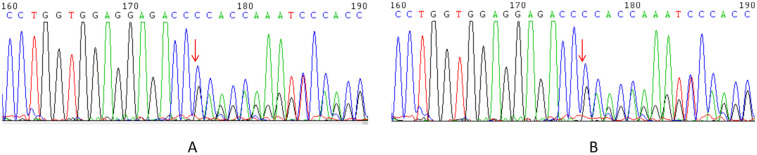
Heterozygous variant in exon 22 of the *TCOF1* gene: TCOF1:NM_001135243.2:c.3559delG (p.Ala1187GlufsTer21). **(A)** The proband. **(B)** The proband's mother.

A heterozygous frameshift variant in exon 22 of *TCOF1* [chr5:g.149772312; *TCOF1*:NM_001135243.2:c.3559delG (p.Ala1187GlufsTer21)] was classified as “Likely Pathogenic” according to the American College of Medical Genetics and Genomics (ACMG) guidelines (PVS1 + PM2_Supporting). PVS1 indicates that this frameshift variant is predicted to cause loss of gene function, while PM2_Supporting indicates that it is absent from population databases. Searches of the Clinical Variant (ClinVar), Human Gene Mutation Database (HGMD), and Genome Aggregation Database (GnomAD) databases, combined with a comprehensive literature review, identified no prior reports of this variant, confirming it as a novel variant. The variant follows an AD inheritance pattern and was maternally inherited. Sanger sequencing confirmed the variant in the proband and his mother (Figure 4). Paternal and maternal grandmother samples were unavailable.

### 2.3 Sanger sequencing

Sanger sequencing was performed to validate the variants identified by whole-exome sequencing. The primers are as follows: F- 5′-GTGCAAGGAGTGTTGAAGCAG-3′, R- 5′-GAGGGATCGGGTAGACAGGAG-3′. The reference sequence NM_001135243.2 of *TCOF1* was used.

## 3 Discussion

TCS is predominantly caused by *TCOF1* variants (88.71% of cases) (7). Over 200 distinct pathogenic variants have been reported globally to date, distributed across exons 3, 5, 6, 10, 12, 13, 15, 16, 18, 20, 22, 23, and 24 (3, 8, 9). Exon 24 is a mutational hotspot region (17.75% frequency). A prototypical variant in this exon (c.4369_4373delAAGAA) induces a frameshift variant that disrupts the nuclear localization signal (NLS), compromising nucleolar localization of the Treacle protein, consequently leading to severe craniofacial malformations (reflected by higher Teber scores) (7, 10).

**Figure 5:**
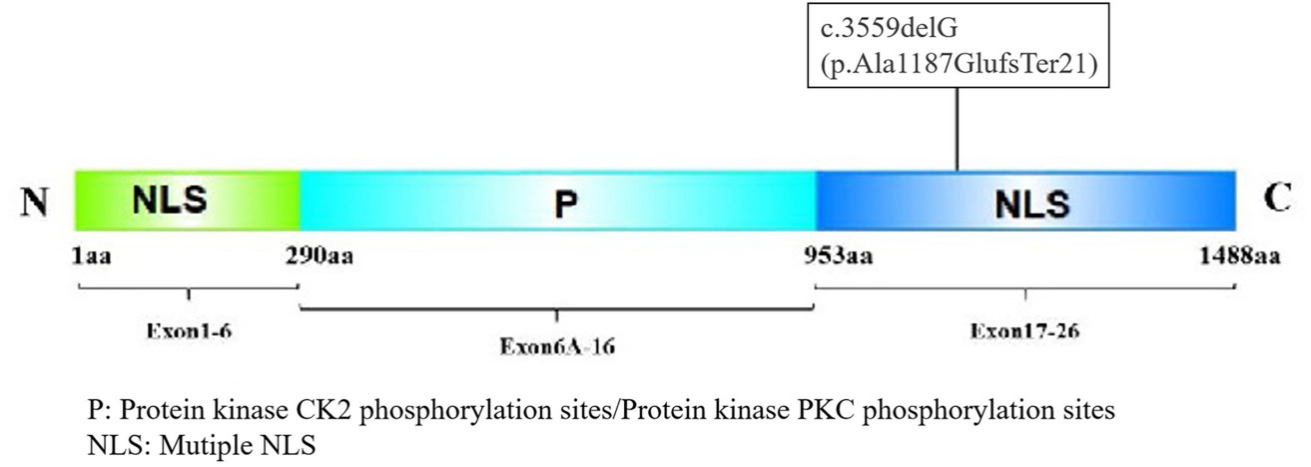
The Treacle protein consists of an N-terminal, C-terminal, and central repeat region. The N-terminal contains the LAQPVTLLDI sequence, which may serve as a nuclear export signal (NES) between 40 and 49 aa, and an NLS between 74 and 77 aa (11). The central structure contains many phosphorylation sites for casein kinase II (CK2) and protein kinase C (PKC). The C-terminus has an NLS, and the phosphorylation site of the Treacle protein by ATM/ATR kinase is located at the Serine-Glutamine/Threonine-Glutamine (SQ/TQ) site of the amino acid sequence (12). The C-terminus contains two SQ sites, at positions 1,199–1,200 and 1,216–1,217 of its amino acid sequence, of which positions 1,199–1,200 are necessary for nucleolar localization.

This study reports a pediatric TCS case harboring a novel *TCOF1* variant, *TCOF1*:NM_001135243.2:c.3559delG (p.Ala1187GlufsTer21), located within exon 22. This deletion induces a frameshift at codon 1,187 (alanine), incorporating 21 aberrant amino acids and subsequent premature termination, resulting in a C-terminally truncated Treacle protein (Figure 5).

This variant exhibits a molecular mechanism analogous to the hotspot variant *TCOF1*:NM_001135243.2:c.4369_4373delAAGAA (p.Lys1457GlufsTer12). It disrupts the function of the critical C-terminal NLS within the Treacle protein. Specifically, the variant ablates the conserved serine residue at position 1199 (S1199), which serves as a phosphorylation target for ATM/ATR kinases (11, 12). Loss of S1199 phosphorylation prevents the treacle protein from localizing to nucleoli, impairing DNA damage response (DDR) activation and the repair of DNA double-strand breaks (DSBs) (13). These defects disrupt ribosome biogenesis and trigger apoptosis in cranial neural crest cells, manifesting as characteristic craniofacial dysmorphology, including malar hypoplasia, mandibular hypoplasia, cleft palate, and ossicular chain malformation (14).

The proband exhibited all typical TCS craniofacial features, plus cardiac malformation (11% incidence) and delayed motor development (1.7%–10% incidence) (3). This broader phenotypic spectrum suggests the following: (1) the premature truncation may impact multiple functional domains of the C-terminal within the Treacle protein; (2) the parents of this proband are consanguineous. Consanguinity elevates autozygosity, thereby increasing the probability that the affected child carries clinically undetected pathogenic variants in additional genes. These variants may engage in synergistic interactions with the *TCOF1* mutation to enhance pathogenicity. This variant provides insight into the *TCOF1* genotype–phenotype correlation and confirms the critical role of the C-terminal NLS domain in Treacle protein function.

Although individuals with TCS typically exhibit characteristic phenotypic features, incomplete penetrance is observed in carriers of pathogenic *TCOF1* variants. The proband's mother, a heterozygous carrier of the c.3559delG variant, was asymptomatic, consistent with previous reports (15, 16). Variable expressivity arises from a multifactorial regulatory network involving the following: (1) protein functional compensation, where the residual N-terminal domain of the truncated Treacle protein may partially sustain ribosome biogenesis through interactions with ribosomal factors such as the DDX21 protein (17); (2) epigenetic modulation, such as DNA hypomethylation activating residual mutant allele transcription (18), while compensatory histone modifications (e.g., H3K27ac enrichment) may upregulate alternative gene expression pathways; (3) environmental stress interference, whereby embryonic oxidative stress may exacerbate phenotypic severity by inducing DNA damage in neural crest cells or degrading *TCOF1*-interacting regulators like *CNBP* (19). The dynamic interplay between genomic integrity, epigenetic landscapes, and environmental stressors poses significant challenges for genetic counseling and prenatal risk assessment.

TCS is a multisystem disorder that requires a multidisciplinary approach beyond surgery. We propose an integrated clinical protocol incorporating the following: (1) rigorous developmental surveillance incorporating serial anthropometry, Gesell Developmental Schedules, and age-stratified auditory brainstem response (ABR) testing to preempt auditory-speech delays; (2) staged craniofacial reconstruction with intervention timing determined by craniofacial 3D CT severity indices, feeding status, and neurodevelopmental milestones. Priority interventions include atresiaplasty for aural atresia, palatoplasty, and computer-aided design/computer-aided manufacturing (CAD/CAM)-guided zygomaticomaxillary complex (ZMC) reconstruction (20); (3) multimodal rehabilitation combining bone-anchored hearing aid (BAHA) devices with evidence-based auditory-verbal therapy (AVT) and orofacial myofunctional therapy (OMT); (4) targeted psychosocial support through structured body-image counseling and peer-mediated social-skills training. This framework simultaneously addresses craniofacial dysmorphology, functional deficits, and psychosocial vulnerability, thus facilitating adaptive self-identity development.

## 4 Conclusions

In conclusion, pathogenic *TCOF1* gene variants are the primary genetic cause of TCS. These alter the encoded amino acid sequence of the Treacle protein, disrupting its phosphorylation and impairing critical functional domains. This dysfunction ultimately leads to aberrant craniofacial chondrogenesis, manifesting as the characteristic facial dysmorphology. The novel variant identified here affects a conserved phosphorylation site in Treacle's C-terminal domain, abolishing NLS function, providing *in vivo* evidence for *TCOF1*'s role in TCS pathogenesis. Incomplete penetrance complicates genetic counseling. This variant expands the mutational spectrum of *TCOF1*, enriching databases critical for future diagnostic and population studies.

## Data Availability

The original contributions presented in the study are included in the article/Supplementary Material, further inquiries can be directed to the corresponding author.
